# Inferior Healing Rate in Isolated Meniscal Repair than that in Meniscal Repair with Concomitant ACL Reconstruction Evaluated with MRI

**DOI:** 10.5704/MOJ.2303.008

**Published:** 2023-03

**Authors:** M Isono, H Koga, Y Nakagawa, T Nakamura, I Sekiya, H Katagiri

**Affiliations:** 1 Department of Joint Surgery and Sports Medicine, Tokyo Medical and Dental University, Tokyo, Japan; 2 Center for Stem Cell and Regenerative Medicine, Tokyo Medical and Dental University, Tokyo, Japan

**Keywords:** meniscus repair, anterior cruciate ligament reconstruction, meniscus tear, healing rate

## Abstract

**Introduction:**

Isolated meniscal repair has been suggested as one of the contributing factors in unhealed meniscal repair. The purpose of this study was to compare the healing rate between isolated meniscal repair and meniscal repair with concomitant anterior cruciate ligament reconstruction (ACLR) using a standardised assessment method after propensity score matching.

**Materials and methods:**

Accuracy of the Crues' grading system for meniscal healing was validated using second-look arthroscopy as the reference standard in 17 patients. Propensity score matching (one-to-one) was performed between 26 patients who underwent isolated meniscal repair and 98 patients who underwent meniscal repair with concomitant ACLR. Patients were matched for sex, age, side and zone of the meniscal repair, and number of sutures. Healing rates at one year which were evaluated with magnetic resonance imaging (MRI) were compared between the two groups.

**Results:**

The sensitivity and specificity of the Crues' grading system on multiple plane MRI for meniscal healing were 100% and 83.3%, respectively. Both the isolated meniscal repair group and the meniscal repair with concomitant ACLR group included 21 patients after propensity score matching. Baseline characteristics did not differ significantly between the two groups. The healing rate was significantly lower in the isolated meniscal repairs group (14.3%) than in the meniscal repair concomitant with ACLR group (47.6%, P=0.04).

**Conclusion:**

The healing rate for isolated meniscal repair using a standardised MRI assessment method was inferior to that of meniscal repair with concomitant ACLR after propensity score matching.

## Introduction

There has been growing interest in arthroscopic repair for management of meniscus tears, in order to prevent early development of osteoarthritis by restoring the native structure and biomechanical properties of the meniscus^1,2^. Post-operative unhealed meniscal repair remains a critical challenge. The risk factors for unhealed meniscal repair have been investigated in terms of tear pattern, avascular zone, and isolated meniscal repair^3^. Several retrospective studies and case control studies have reported isolated meniscal repair to be one of the predictive risk factors for unhealed meniscal repair compared to meniscal repair concomitant with anterior cruciate ligament reconstruction (ACLR)^4-6^. However, retrospective studies and case control studies are associated with methodological weaknesses regarding selection bias and distribution of confounding factors between exposure groups and control groups. Propensity score matching ensures that the distribution of measured baseline confounding factors are similar in exposure and control groups and reduces the effects of confounding factors when analysing observational data^7^.

Post-operative assessment of meniscal repairs—healed or unhealed— is based predominantly on magnetic resonance imaging (MRI) evaluation using the Crues' grading system. The Crues' grading system is the gold standard method for diagnosing meniscus tears and evaluates the presence of an area of increased signal intensity on MRI^8^. However, the reported accuracy of the Crues' grading system for diagnosis of recurrent tears after meniscus repair ranges widely, from 57% to 80%, due to methodological differences^9^. Therefore, validation of the accuracy of the Crues' grading system for diagnosing recurrent tear is urgently needed.

The principal purpose of this study was to compare the healing rate of meniscal repair evaluated using the standardised assessment method in patients who underwent isolated meniscal repair and patients who underwent meniscal repair concomitant with ACLR using propensity score matching. The hypothesis underlying this study was that the healing rate of meniscal repair alone would be poor in comparison to meniscal repair with ACLR. Prior to the principal study, the accuracy of the Crues' grading system for diagnosis of recurrent tears was validated using second-look arthroscopy as the reference standard.

## Materials and Methods

The present study was designed to compare the short-term outcomes of meniscal repair with and without ACLR in a two-stage model. In the first stage, in order to validate MRI as a diagnostic tool for recurrent tear, MRI findings and second-look arthroscopy findings after meniscal repair were compared. In the second stage, as the first purpose of the study, the two groups were matched using propensity score matching and compared regarding MRI findings. This study was approved by the Institutional Review Board of the authors’ affiliated institutions. All study participants provided their full written informed consent for participation in this clinical research prior to undergoing the operative procedure.

Four hundred and twenty-five patients who underwent primary arthroscopic meniscal repairs between January 2011 and December 2018 at our institute were identified. In the first stage, patients who met the following inclusion criteria were included: (1) patients who agreed to undergo second-look arthroscopy; (2) patients with no additional injury on the operated knee joint; and (3) patients who had undergone MRI before second-look arthroscopy. In the second stage, patients who underwent primary meniscal repair and met the following inclusion and exclusion criteria were included in the propensity score matched analysis. The inclusion criteria were: (1) patients who had undergone MRI at a one-year follow-up; (2) patients with no additional injury on the operated knee joint; and (3) (for the ACLR group) patients who underwent primary ACLR conducted with a hamstring tendon autograft. The exclusion criterion was patients who had undergone both medial and lateral meniscal repair. Propensity score matching was used to control for potential selection bias. Propensity score matching (one to one) was performed between the two groups (meniscal repairs concomitant with ACLR group and isolated meniscal repairs group) in terms of sex, age, side of the meniscal repair (medial or lateral), zone of the meniscal repair (white-white, white-red, red-red), and number of sutures. The average, standardised average, and average ranks of included variables were compared between the two groups in the propensity score matching process.

All surgeries were performed by two senior attending surgeons or under their supervision. Meniscal injuries were managed according to the injury status. Meniscal tears were repaired using the all-inside suture technique, using the FasT-Fix device [Smith and Nephew, Andover, MA, USA] and/or the inside-out suture technique, using the Henning meniscal suture kit [Stryker, Kalamazoo, MI, USA]. Mostly, the inside-out technique was applied for middle to posterior segments, and the all-inside technique was applied for posterior segments. The number of sutures was dependent on the size of the tear. As previously described, an anatomic double-bundle technique using an autologous semitendinosus tendon was employed for ACLR^10^. Briefly, the semitendinosus tendon was harvested, cut into halves, and folded, making two double-stranded bundles. Both tibial and femoral tunnels were drilled at the anatomic insertion sites of each bundle: the anteromedial bundle and posterolateral bundle. The femoral sides of the grafts were anchored with an Endobutton CL-BTB [Smith and Nephew] and the tibial sides of grafts were anchored with two anchor staples.

The post-operative rehabilitation protocol was the same for all patients regardless of whether or not concomitant ACLR was performed. One day after surgery, patients started range of motion exercises and walking exercises with the help of a knee immobiliser, though the use of crutches was permitted. Crutches were used for six weeks. Running exercises was allowed around three months post-procedure. Patients, who recovered sufficient muscle strength were permitted to partake in full athletic activities six months after surgery.

Second look arthroscopy was performed patients in with symptoms of swelling knee or undergoing staple removal. Staple removal was conducted in the absence of symptoms. Second-look arthroscopy was performed by senior attending surgeons or under their supervision. The repaired meniscus was judged as healed using the following criteria: (1) smooth surface of the repaired meniscus on the femoral and tibial sides and (2) good stability of the repaired meniscus when pulled with a probe. Otherwise, we judged the meniscus to be unhealed^11^.

**Fig. 1:**
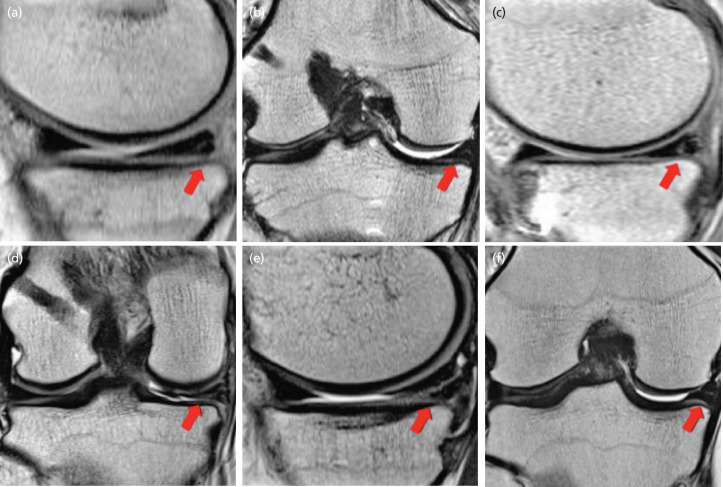
Crues’ grading system using magnetic resonance imaging (a, b) Grade 1 in sagittal and coronal view. No abnormal signal is seen on the meniscus. (c, d) Grade 2 in sagittal and coronal view. Red arrows indicate linear signal without communication with an articular surface. (e, f) Grade 3 in sagittal and coronal view. Red arrow indicates linear signal communicating with articular surface.

The MRI scans were performed using a 3-T MRI system [Magnetom Verio, Siemens Medical Systems, Erlangen, Germany]. The following sequences were obtained: (1) sagittal T2-weighted spin echo: repetition time (TR) 4300ms, echo time (TE) 83mm and (2) coronal T2-weighted spin echo: TR 4300ms, TE 83mm thickness 4mm, and space, 1mm. The direction of coronal scans was made parallel with the line of the medial and lateral femoral condyles, while the sagittal scans were vertical. In the MRI scans, the signal grade at the repaired site was assessed using the Crues’ criteria on the MRI by an orthopaedic specialist who did not perform the surgery in this series and who was blinded to the arthroscopic evaluation on meniscus healing^8^. The signal grade at the repaired site was assigned as follows: grade 1: an irregularly marginated intrameniscal signal was shown, without abutting or communicating with an articular surface (Fig. 1a, 1b) grade 2: a linear signal was shown, without abutting or communicating with an articular surface (Fig. 1c, 1d) grade 3: a similarly linear signal intensity was shown, but extending to the articular surface, whether that be to the tibial or to the femoral site (Fig. 1e, 1f). The signal grade at the repaired site was graded using sagittal and coronal T2-weighted spin echo images, respectively. Multiple plane grading was defined as the higher grade of the two planes. Grades 1 and 2 signals found at the repaired site were interpreted as a healed meniscus, and a grade 3 signal as an unhealed meniscus^8^. The MRI and second-look arthroscopy findings, which was used as the reference standard, were compared. The sensitivity, specificity, accuracy, positive predictive value, and negative predictive value were calculated for MRI findings of each plane. The intra-reader intraclass correlation coefficient (ICC) for MRI grading of the repaired site was determined using assessments performed on different days by a single orthopaedic specialist using the MR images from the first stage. There was an interval of >4 weeks between the first and second assessments. The inter-reader ICC for MRI grading of the repaired site was determined using assessments conducted by two independent examiners using the MR images from the first stage.

Knee instability was examined via physical examination with Lachman's test, the pivot shift test, and the anterior drawer test at the one-year follow-up. All examinations were conducted by an orthopaedic specialist.

All statistical analyses, except the post-hoc power analysis, were performed using the EZR software (Jichi Medical University, Japan), which is a modified version of R commander that is designed to add biostatistical functions^12^. In the second step, differences in age, height, weight, BMI, and number of sutures in the ACLR group and the isolated meniscal repair group were assessed using a Student`s t-test, after confirming the normality using the histogram, and the assumption of equal variance was examined using the F test. Differences in sex, side of the meniscus, zone of the meniscus, location of the meniscus, time from injury to suture, the result of physical exam, and the healing status between the two groups were assessed using the Fisher exact test. A P-value less than 0.05 was considered statistically significant. Data were expressed as mean with 25% and 75% percentiles. Post-hoc power analyses for the Fisher exact test of healing status were performed using G*Power 3.1 [UCLA; California, USA]^13^.

## Results

Patient characteristics are shown in (Table I). Among the 17 repaired menisci, six menisci were completely healed, representing a total healing rate of 35%. The rate of grade 3 signals was 47% in the sagittal plane and 53% in the coronal plane. In multiple plane MRI evaluations, the rate of grade 3 signals was 71%. The multiple plane MRI grading showed high sensitivity and high accuracy when second-look arthroscopy assessment was used as the reference standard (Table II). The intra-reader ICC for Crues’ grading was 0.87 and the inter-reader ICC was 0.76. Therefore, the results were considered to be excellent.

**Table I:** Patient characteristics and intra-operative data (first stage)

Patient characteristic	
Number of patients	17
Age (years), mean (range)	19 (17–25)
Sex, Female / Male n (%)	10 (58.8) / 7 (41.2)
Height (cm), mean (range)	164 (160–172)
Weight (kg), mean (range)	59 (57–65)
BMI, mean (range)	22.5 (21.3–24.1)
with ACLR, n (%)	13 (76.5)
**Time from injury to suture, n (%)**	
<3 months / ≥3 months	7 (41.1)/ 10 (58.8)
Time from suture to MRI (months, mean (range)	12.3 (7.8–20.0)
Time from suture to second-look arthroscopy (months), mean (range)	20.5 (15.6–26.4)
**Intra-operative Data**	
Meniscus side, medial / lateral n (%)	10 (58.8) / 7 (41.2)
Location of meniscal tear, n (%)	
Posterior segment	6 (35.3)
Middle to posterior segment	10 (58.8)
Anterior to posterior segment	1 (5.9)
Meniscus tear zone, n (%)	
Red-red / White-red / White-white	4 (23.5) / 9 (52.9) / 4 (23.5)
Meniscus tear pattern, n (%)	
Vertical/ Horizontal/ Raidal/ Complex/ Bucket hundle	5 (29.4)/ 4 (23.5)/ 2 (11.8)/ 1 (5.9)/ 5 (29.4)
Meniscus repair procedure, n (%)	
All inside / Inside-out / All inside and Inside-out	8 (47.1) / 1 (5.9) / 8 (47.1)
Number of sutures, median (range)	3 (1–8)
Healed meniscus by second-look arthroscopy, n (%)	6 (35.3)

Notes: Values with brackets are expressed as mean with 25% and 75% percentiles. Values in parentheses are expressed as numbers with percentages.

Abbreviations – BMI: body mass index, MRI: magnetic resonance imaging, ACLR: anterior cruciate ligament reconstruction

**Table II:** Diagnostic results (in percentages) by MRI plane

Plane	Sensitivity, (%)	Specificity, (%)	Accuracy, (%)	PPV, (%)	NPV, (%)
Coronal T2	72.7	100.0	82.4	100.0	66.7
Sagittal T2	72.7	83.3	76.5	88.9	62.5
Multiple plane	100.0	83.3	94.1	91.7	100.0

Abbreviations – MRI: magnetic resonance imaging, PPV: positive predictive value, NPV: negative predictive value

**Fig. 2:**
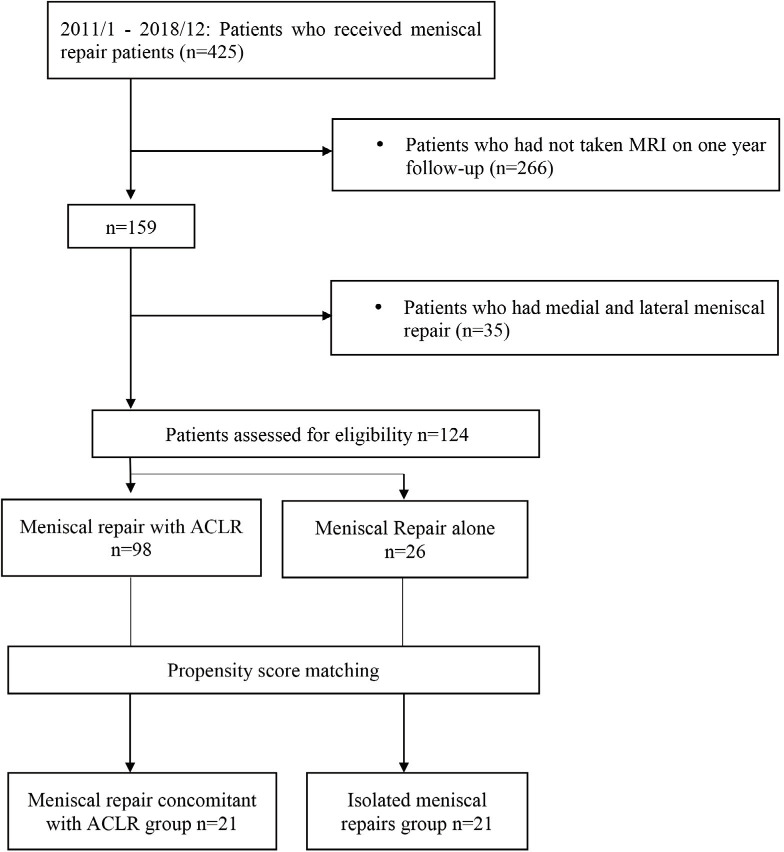
Flowchart showing selection of patients for analyses in the second stage.

In the present trial, 98 patients who underwent meniscal repair and concomitant ACLR and 26 patients who underwent isolated meniscal repair were eligible for inclusion. Propensity score matching was used to control for potential selection bias. Both groups comprised 21 patients (Fig. 2). The baseline characteristics were not significantly different in the two groups, including items matched by propensity score matching and items not matched by propensity score matching (height, weight, BMI, time from injury to surgery: (Table III). The two groups were comparable in terms of knee instability. The meniscus healing rate was significantly higher in the meniscal repair and concomitant ACLR group than in the isolated meniscal repair group [odds ratio: 5.5 (95% confidence interval 1.2– 24.3); (Table IV). Post-hoc power analysis showed that, with an alpha of 0.05, a sample size of 21 cases in each group obtained a power of 0.68 for differences in healing rate between the concomitant ACLR group and the isolated meniscal repair group.

**Table III:** Patient characteristics of matched groups (second stage).

Patient characteristic	ACLR group (n=21)	Isolated group (n = 21)	P value
Age (years), mean (range)	21 (17–32)	28 (19–36)	ns
Sex, Female / Male n (%)	10 (47.6) / 11 (52.4)	10 (47.6) / 11 (52.4)	ns
Height (cm), mean (range)	164 (161.6–168)	167 (161–170)	ns
Weight (kg), mean (range)	61 (58–65)	62 (55–67)	ns
BMI, mean (range)	22.7 (21.8–23.9)	21.9 (20.4–23.9)	ns
Meniscus side, Medial / Lateral n (%)	13 (61.9) / 8 (38.1)	15 (71.4) / 6 (28.6)	ns
Number of sutures, mean (range)	9.5 (3.3–11)	6 (4–9)	ns
Tear zone, n (%)		ns	
Red-red / White-red / White-white	3 (14.3) / 8 (38.1) / 10 (47.6)	4 (19.0) / 9 (42.3) / 8 (38.1)	
Location of meniscal tear, n (%)			ns
Posterior segment 5 (23.8)	4 (19.0)		
Middle to posterior segment	15 (71.4)	12 (57.1)	
Anterior to posterior segment	1 (4.8)	5 (23.8)	
Meniscus tear pattern, n (%)			
Vertical/ Horizontal/ Raidal/ Complex/	9 (42.3)/ 2 (9.6)/ 1 (4.8)/	9 (42.3)/ 3(14.4)/ 2(9.6)/	ns
Bucket hundle	5 (23.8)/ 4 (19.0)	3 (14.4)/ 3(14.4)	
Time from injury to suture, n (%)			ns
<3 months / ≥3 months	9 (42.9) / 12 (57.1)	11 (52.4) / 10 (47.6)	
Time from suture to MRI(months), mean(range)	12.0 (11.5–12.8)	11.8 (9.3–12.3)	ns
Knee instability test			
Lachmann, n (%)	2 (9.5)	0	ns
ADT, n (%)	1 (4.8)	0	ns
Pivot shift test, n (%)	3 (14.3)	1 (4.8)	ns

Notes: Values with brackets are expressed as mean with 25% and 75% percentiles. Values in parentheses are expressed as numbers with percentages.

Abbreviations – BMI: body mass index, ADT: anterior drawer test, ACLR: anterior cruciate ligament reconstruction

**Table IV:** Healing rate of repaired menisci evaluated with MRI

	ACLR group	Isolated group	P value
Healing rate (%)	47.6	14.3	0.04*

Abbreviations – MRI: magnetic resonance imaging, ACLR: anterior cruciate ligament reconstruction

## Discussion

The most important finding of the present study was that when the standardised MRI assessment method was used, the healing rate of isolated meniscal repair was inferior to that of meniscal repair with concomitant ACLR after propensity score matching. Prior to the principal study comparing isolated meniscal repair and meniscal repair with concomitant ACLR, accuracy of the Crues' grading system using multiple plane MRI for meniscal healing was validated using second-look arthroscopy as the reference standard. The Crues' grading system using multiple plane MRI showed good diagnostic performance in terms of the meniscal healing status. Therefore, in the principal study, the Crues' grading system and multiple plane MRI were used as the standardised assessment methods.

According to the current body of literature on MRI outcomes of meniscal repair with concomitant ACLR, a wide range of meniscus healing rates have been reported from 34% to 83% of the cases^14-19^. Healing rates of suture repairs for isolated meniscal tears range between 4% and 50%^20-22^. The healing rates of meniscal repair for both isolated repair and repair with concomitant ACLR varied greatly among studies since the criteria of meniscus healing assessment with MRI are not uniform and difficult to compare. In this study, the healing rate was 47.6% for ACLR and 14.3% for the isolategroup which were in the same range as previous studies, we were able to compare between the two groups using the same meniscus healing criteria on MRI. One systematic review showed a higher re-operation rate for isolated meniscal repair compared to meniscal repair with concomitant ACLR (24% vs. 14%, respectively)^23^. Another study showed a higher re-operation rate at 2 years for isolated meniscal repair compared to meniscal repair with concomitant ACLR (16.7% vs. 9.7%, respectively)^24^. However, the re-operation data for both aforementioned studies were obtained via electronic medical records and did not include the healing status of the treated menisci. It is important to consider that clinical success without re-operation does not necessarily imply complete healing of the lesion and mechanical strength. It has been argued that the definition of clinical success includes patients with incomplete healing and future re-tears, and therefore, poor outcomes are underestimated^25^. The present study assessed the healing status of meniscal repairs using strict criteria on MRI.

Healing of meniscal repairs with concomitant ACLR was better than that with isolated meniscal repair, in this study. Age, side, zone, and size of injured meniscus, the time from injury to repair, and concomitant augmenting techniques may affect the healing of meniscal repairs^4,26^. In the present study, the two groups were matched for these factors using propensity score matching, except for concomitant ACLR. Therefore, the result might be affected by the difference in the procedure, namely concomitant ACLR. In an animal model, concomitant marrow venting significantly improved the healing rate for meniscal repair^27^. It is thought that growth factors and stem cells, from bone marrow at an ACL tunnel for graft, enhance the biological environment and meniscal repair at the repair site^28^. This theory has been applied to augmenting techniques of venting marrow procedures and perforations at the intercondylar notch in the treatment of isolated repairs^29,30^.

Post-operative arthroscopy is not recommended for all patients because it is an invasive procedure. Several studies have addressed the sensitivity of MRI for meniscus status after meniscus repair, compared with the use of second-look arthroscopy as the reference standard. The reported sensitivity for diagnosing recurrent tears after meniscus repair ranged from 75% to 92% for single plane MRI^11,31^, and from 79% to 87% for multiple plane MRI^8,32^. In line with the previous studies, both the Crues' grading system using multiple plane MRI and single plane MRI showed good diagnostic performance for meniscal healing status in the present study. Miao *et al* searched and calculated MRI in five sequences and showed different sensitivities according to the plane of the MRI image^11^. This result suggests that one sequence of the image might hide the parallel injury; therefore, using the sagittal and coronal images might improve the accuracy and sensitivity for diagnosing the healing status of treated meniscus using MRI.

There were some limitations to the present study. First, the number of patients in the two groups was small, which affected the evaluation of diagnostic value. In further studies, the total number of patients needs to be increased. Second, when matching the two cohorts, we used age, side, zone, and size of the meniscus as factors that influenced the result of the meniscal repair in our study. Lower limb alignment was not measured which might affect the healing rate and it is possible that other factors that affect healing were overlooked. Third, several patterns of meniscal injury were evaluated overall in this study. In further studies, evaluation should be conducted with each pattern of meniscal injury. Fourth, our results reflect outcomes at the one-year postoperative follow-up, and cannot inform long-term results; however, if a meniscus is not healed at one year, it is unlikely to do so. Fifth, the inferior result of meniscal repair alone might relate to occult instability. Knee instability is supposed to increase the possibility of failure of meniscal repair^33^. The measured stability was the same in the two groups, but there was a possibility that some of these knees had an occult undetectable instability, and that this occult instability attributed to the results. Sixth, in this study we could affect the repaired meniscus by Crues' grading system using multiple plane MRI. Instead of assessing the repaired meniscus with arthroscopy several less invasive examination tools such as Crues' grading system were used, though there is no consensus on the gold standard method. Seventh, since only patients who agreed to second-look arthroscopy were included in the first stage, there was inclusion bias in the first stage study. Lastly, the interval from meniscal repair to second-look arthroscopy was not standardised.

The clinical relevance of this study is that isolated meniscal repair is one of the risk factors for unhealed meniscus. Therefore, in cases of isolated meniscus repair, additional augmentations such as bone marrow stimulation, fibrin clot, and regenerative therapies might be considered.

## Conclusion

The healing rate for isolated meniscal repair as assessed by a standardised MRI method was inferior to that of meniscal repair with concomitant ACLR after propensity score matching.
